# Learning Molecular Representation in a Cell

**Published:** 2024-06-22

**Authors:** Gang Liu, Srijit Seal, John Arevalo, Zhenwen Liang, Anne E. Carpenter, Meng Jiang, Shantanu Singh

**Affiliations:** 1 University of Notre Dame; 2 Broad Institute of MIT and Harvard

## Abstract

Predicting drug efficacy and safety *in vivo* requires information on biological responses (e.g., cell morphology and gene expression) to small molecule perturbations. However, current molecular representation learning methods do not provide a comprehensive view of cell states under these perturbations and struggle to remove noise, hindering model generalization. We introduce the **Info**rmation **Align**ment (InfoAlign) approach to learn molecular representations through the information bottleneck method in cells. We integrate molecules and cellular response data as nodes into a context graph, connecting them with weighted edges based on chemical, biological, and computational criteria. For each molecule in a training batch, InfoAlign optimizes the encoder’s latent representation with a minimality objective to discard redundant structural information. A sufficiency objective decodes the representation to align with different feature spaces from the molecule’s neighborhood in the context graph. We demonstrate that the proposed sufficiency objective for alignment is tighter than existing encoder-based contrastive methods. Empirically, we validate representations from InfoAlign in two downstream tasks: molecular property prediction against up to 19 baseline methods across four datasets, plus zero-shot molecule-morphology matching.

## 1 Introduction

Drug properties, e.g., toxicity and adverse effects [25], are induced by molecular initiating events—interactions between a molecule and a biological system—that first impact the cellular level and ultimately influence tissue or organ functions [31, 25]. However, a chemical molecule’s structure alone is insufficient information to predict its impact on cells: each chemical interacts with multiple cells and genes and induces complex changes in gene expression and cell morphology, making predictions of downstream responses challenging [5, 49, 32]. Hence, *molecular representation learning should make use of information about cellular response*, enhancing the representation of the mode of action and thereby improving predictions for downstream bioactivity tasks [25, 53].

There is a lack of exploration for holistic molecular representations from molecular structure, cell morphology, and gene expression [17, 57, 26, 53, 45]. For example, graph self-supervised methods only manipulate molecular structures to perturb or mask molecular graphs using contrastive or predictive losses [17, 57, 20]. Moshkov et al. [32] explored the ability of different data modalities, taken independently, to predict molecules’ assay activity in a diverse set of assays (tasks). They found (from [32]’s Fig.2) that molecular structure supports highly accurate prediction (AUC > 90%) in 31% (16/52) of tasks, gene expression in 37% (19/52) and cell morphology in 54% (28/52). Similarly, in our experiments (Figure 3), we observe that molecular structure is not a one-size-fits-all solution.

**Figure 1:**
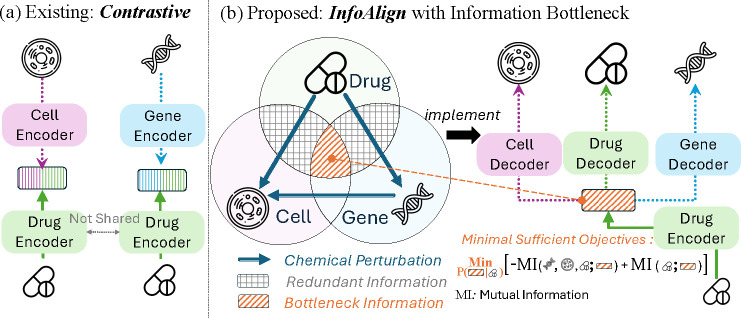
Molecular Representation Learning via the Information Bottleneck: (a) Existing contrastive learning methods utilize two encoders—one for molecules and another for cell morphology or gene expression features, lacking a holistic view of molecular representation learning in cells. (b) In contrast, InfoAlign excludes redundant information in molecule-cell morphology or molecule-gene expression contrastive pairs that could impair generalization [53] for bottleneck representation.

Cells can be perturbed by treating them with chemicals or genetic reagents that disrupt a particular gene or pathway. These chemical and genetic perturbations *in vitro* naturally bridge molecules with cell morphology and gene expression, as illustrated in Figure 1 (b). However, multi-modal contrastive methods such as CLOOME [45] and InfoCORE [53], depicted in Figure 1 (a), focus primarily on aligning molecular representations with cell morphology [45, 53] or gene expression [53]. These approaches fall short in two ways. (1) They do not remove redundant information, grey-colored area in Figure 1 (b), that may harm representation generalization. The presence of redundant information [53] may induce spurious correlations, adversely affecting the generalization of molecular representations. For example, in small molecule perturbations [3, 6], batch identifiers can signify confounding technical factors, creating misleading associations between molecular structures and cell morphology [53]. (2) They treat molecules as the sole connectors between gene expression and cell morphology, ignoring the potential for genetic perturbations [6] to directly establish connections between these modalities. The data derived from genetic perturbations [6] not only enriches the feature space of gene expression and cell morphology but also enhances the navigation of molecular representation learning towards the overlapped (bottleneck) area in Figure 1 (b).

To address the aforementioned challenges, we conceptualize the cellular response processes as a context graph, capturing a more complete set of interactions among molecules, gene expression, and cell morphology. Using the context graph, we identify neighborhoods for each molecule and apply the information bottleneck [52] to optimize molecular representations, which aligns them with neighboring biological variables to remove redundant information and improve generalization.

We propose the **Info**rmation **Align**ment (InfoAlign) approach, as presented in Figure 1 (b). InfoAlign uses one encoder and multiple decoders with information bottleneck for minimal sufficient statistics in representation learning. The minimality objective optimizes the encoder to learn the *minimal* informative representation from molecular structures by discarding redundant information. The sufficiency objective ensures the encoder retains *sufficient* information, allowing decoders to reconstruct features for biological variables in neighborhood areas of the context graph. We construct the context graph based on molecule and genetic perturbations [4, 6, 49] and introduce more biological (gene-gene interaction [16]) and computational (cosine similarity) criteria to increase edge connectivity. We conduct random walks on the context graph, beginning with the molecule in the training batch, to identify its neighborhood. Cumulative edge weights indicate similarity between the molecule and variables along the path. The molecule is encoded, and its latent representation is decoded to align with features identified in the random walk. Encoders and decoders are jointly optimized using an upper bound for the minimality objective and a lower bound for the sufficiency objective.

The sufficiency objective introduces a decoder-based bound for multi-modal alignment using decoders. We demonstrate the theoretical advantages of this approach, showing it provides a tighter bound than the encoder-based bound used in previous contrastive methods [35, 39, 53, 45] in Section 4.3. In experiments, InfoAlign outperforms up to 19 representation learning baselines across three classification and one regression datasets, with overall improvements of +10.58% and +6.33% on the Broad6K classification and Biogen3K regression datasets, respectively. InfoAlign also exhibits highly competitive zero-shot multi-modal matching capabilities on two molecule-morphology datasets.

## 2 Related Work

### Representation Learning on Molecular Structure:

Representation learning approaches for molecules can be categorized into sequential-based [24, 44] or graph-based models [17, 57, 58, 27]. Sequential models, utilizing string formats of molecules like SMILES and SELFIES [24], have evolved from Recurrent Neural Networks (RNNs) to Transformers [7, 44]. These models typically follow specific pretraining strategies similar to language models such as BERT [9], RoBERTa [30, 7] and GPT [38]. The pretraining targets are thus often the next token predictions or mask language modeling [9, 7] on SMILES or SELFIES sequences [38]. Graph Neural Networks (GNNs) are the architectures for graph-based approaches [17, 57, 58, 29], where methods to pretrain GNNs often perturb or mask the atoms, edges, or substructures of molecular graphs with contrastive [17, 57] and predictive losses [58, 20]. Recent evidence highlights the challenges of developing universal molecular representations based solely on molecular structures without integrating domain knowledge [3, 46, 50, 47, 28]. Although using motifs is a common method to incorporate such knowledge [43, 20], the incorporation of information about molecules’ biological impacts is much less explored. We aim to enhance molecular representation learning by incorporating domain knowledge from cellular response data.

### Representation Learning with Different Modalities:

Existing methods on multimodal alignment, such as CLIP [39], primarily address pairwise relationships between texts and images and use methods like InfoNCE [35, 53, 45]. These approaches use separate encoders for different modalities to compute contrastive loss, which is upper bounded by the number of negative examples [37]. Subsequent research on molecules similarly focuses on pairwise alignment between molecules and cell images [45, 53], molecules and protein sequences [19], and molecules and text [10, 21]. Although BioBridge [55] handles multiple modalities, it leverages a knowledge graph for transforming representations between modalities rather than optimizing molecular representations.

### Representation Learning with Cellular Response Data:

A primary goal of molecular representation learning is to predict molecular bioactivity. Likewise, emerging gene expression [49] and morphological profiling approaches [5, 3, 6, 48] that describe perturbed genetic or cellular states in cell cultures can also be used to predict bioactivity. In some datasets, molecules are the perturbations, and the perturbed cell states measured are gene expression values for a thousand or more genes [49] and/or microscopy Cell Painting images, which can be represented as a thousand or more morphology features [8]. Recently created large-scale perturbation datasets [3, 49, 6] could enrich molecular representation learning approaches. CLOOME [45] and MoCoP [33] contrast cellular images with molecules and InfoCORE [53] contrasts molecule with either morphological profiling [4] or gene expression [53]. InfoCORE [53] aims to mitigate confounding batch identifiers, but its effectiveness depends on a batch classifier, which is impractical without batch identifiers. We integrate cellular response data and molecules into a context graph to capture cellular response patterns, focusing on learning molecular representations to remove nuisances [51].

## 3 Problem Definition

Suppose a molecule X∈𝒳 is from the space 𝒳 constructed by atoms and bonds. An encoder with parameter θ could map X into a D-dimensional representation space $𝒵:f_{\theta }\;:𝒳\to 𝒵\subset R^{D}$. The encoder $f_{\theta }$ could be learned with Graph Neural Networks (GNNs) [23, 56], where atoms are nodes and bonds are edges. Such GNNs first update node representations and then perform a readout operation (e.g., summing) over nodes to obtain the latent representation variable Z∈𝒵 for X.

Existing research in contrastive or generative learning has extensively leveraged structural features to pretrain the GNN encoder $f_{\theta }$ [17, 20, 28]. However, using more expressive features from the cellular context, such as cell morphology C and gene expression E, remains largely unexplored for improving molecular representations.

## 4 The Proposed InfoAlign

**Figure 2:**
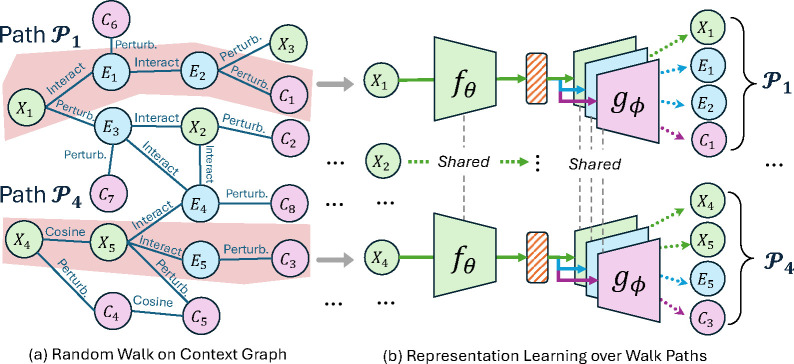
Representation Learning Over Walk Paths in Context Graphs: (a) In Section 4.1, we construct the graph with various interaction, perturbation, and cosine similarities among molecules X, cell morphology profiles C, and gene expression profiles E. Given a training batch of molecules, including $X_{1}$ and $X_{4}$, random walk extracts paths, for instance, of length 4. (b) In Section 4.2, we aim to learn bottleneck representations from molecules with encoder $f_{\theta }$ that minimally relate to structures yet maximally reconstruct feature with decoders $g_{\phi }$ about variables along the walk paths.

We present the overall representation learning framework in Figure 2. In Section 4.1, we construct the context graph for cellular response data. In Section 4.2, we introduce representation learning methods based on the principle of minimal sufficiency for molecules and their related modalities. In Section 4.3, we demonstrate the theoretical advantages of the proposed method.

### 4.1 Random Walks on Cellular Context Graph

In the context graph, molecules, cell morphology, and genes with expression values serve as nodes with feature spaces ${𝒴}^{\left[X\right]},\;{𝒴}^{\left[C\right]}$, and ${𝒴}^{\left[E\right]}$ for vectors and scalars, respectively. Molecular features are vectors obtained using fingerprint [42]. Cell morphology features are vectors derived from CellProfiler [5] applied to Cell Painting microscopy images. Gene expression features are scalars using L1000 [49] methods. We further rescale the feature spaces to a range between 0 and 1.

We link nodes using various chemical, biological, and computational criteria. For example, molecules can perturb cultured human cells, inducing changes in cell morphology [6] and gene expression [49], thus linking them to cell morphology and gene expression nodes. Genes could also perturb cells, inducing links between genes and cell morphology [6]. Additionally, we calculate cosine similarity within the same feature space 𝒴 and use biological criteria such as gene-gene interactions [16] to enrich the edge space. Formally, we define:

#### Definition 4.1 (Cellular Context Graph).

The graph G=(𝒱,ℰ) comprises nodes v∈𝒱, each denoting a data point and mapped to a feature space $\psi \;:𝒱\to \{{𝒴}^{\left[X\right]},{𝒴}^{\left[C\right]},{𝒴}^{\left[E\right]}\}$. Each edge e∈ℰ has a weight from 0 to 1, indicating the similarity between nodes based on specific criteria.

We provide more details on the data sources, node features, and edge weights for constructing the context graph in Section 5.1.

#### Path Extraction with Random Walk

Suppose the molecule X from the training batch corresponds to the node $v_{1}\in 𝒱$ on the cellular context graph. We then extract nodes that have similar patterns as induced by X through random walks. Specifically, we use standard random walks with degree-based transition probabilities [36] and denote the resulting walk path for a given molecule as ${𝒫}_{X}$. This path is an L-length sequence: $X=v_{1}\overset{e_{1}}{\to }\ldots \overset{e_{L}}{\to }v_{L}$. The cumulative product of edge weights to node $v_{i}\;(1\leq i\leq L)$, denoted as $\alpha \left(v_{i}\right)=\prod_{j=1}^{i}e_{j}$, quantifies the similarity between X and the node $v_{i}$.

### 4.2 Optimization for Bottleneck Representation

We aim to extract minimal sufficient information from X through aligning its representation with features from variables along the walk path ${𝒫}_{X}$, which could effectively mitigate nuisance [51]. To achieve this, we define the bottleneck representation of molecule X using two mutual information (MI) principles: (1) the minimality principle, which minimizes MI between molecules and their latent representations as I(X;Z), and (2) the sufficiency principle, which decodes latent representations to maximally reconstruct feature spaces for variables along the walk path $\sum_{v\in {𝒫}_{X}}I(Z;\psi (v))$. Together, these form the minimal sufficient optimization objectives:

(1) $$\underset{p(Z|X)}{\min}[-\sum_{v\in {𝒫}_{X}}I(Z;\psi (v))+\beta I(X;Z)],$$

where β controls the tradeoff between minimality and sufficiency. The exact computation of I(Z;ψ(v)) and I(X;Z) is intractable due to the unknown conditional distribution p(ψ(v)∣Z) and the marginal p(Z). We introduce the variational approximations q(ψ(v)∣Z) and q(Z) for them, respectively. This results in a lower bound estimation for the first decoding term $I_{DLB}$ and an upper bound for the second encoding term $I_{EUB}$ [37].

(2) $$I(Z;\psi (v))\;\geq E_{p(Z,\psi (v))}[log\;q(\psi (v)|Z)]+H(\psi (v))≜I_{DLB}\;\;I(X;Z)\;\leq E_{p(X)}[KL(p(Z|X)∥q(Z))]≜I_{EUB}$$

H(ψ(v)) is the differential entropy. Proofs are in appendix A.1. Combined together, $I_{DLB}$ and $I_{EUB}$ upper bound the Eq. (1) to form a tractable objective $-I_{DLB}+I_{EUB}$ to optimize the encoder $f_{\theta }$ with different feature decoders $g_{\phi }^{\left[X\right]},\;g_{\phi }^{\left[E\right]},\;g_{\phi }^{\left[C\right]}$ for molecular features, gene expression features, and cell morphology features, respectively. Specifically, the encoder $f_{\theta }(X)$ learns the mean and variance $\mu_{Z}\in R^{D}$, $\sigma_{Z}\in R^{D}$ of the latent variable Z. The decoder $g_{\phi }$ could take as inputs the mean to reconstruct features for the neighboring variables of X on the context graph. After ignoring the constant terms, we could implement Eq. (2) as the loss function for the molecule X:

(3) $$L=\frac{1}{L}\sum_{v\in {𝒫}_{X}}\alpha (v)\;\text{CE}\left(g_{\phi }^{\left[v\right]}\left(f_{\theta }(X)\right),\psi (v)\right)+\beta \;\text{KL}\left(f_{\theta }(X)\|N(0,I)\right),$$

where CE and KL are cross entropy and Kullback–Leibler divergence, respectively. The Gaussian N(0,I) is the prior distribution [1]. With reparameterization [22], we can directly backpropagate through the neural network to optimize parameters θ and ϕ.

InfoAlign uses multiple decoders $g_{\phi }$ to align multi-modal features, while prior work relies on multi-encoders with CLIP-like losses to align the latent space [39, 14, 53, 45]. Next, we provide the theoretical benefits of decoder-based alignment alongside the empirical advantages in Section 5.

### 4.3 Theoretical Motivation for Decoder-based Alignment

InfoNCE [35] is the contrastive loss used for most CLIP-like methods [39, 53]. In this work, we show that the mutual information lower bound based on InfoAlign is tighter than that based on InfoNCE.

#### Proposition 4.2.

*For tthe molecular representation* Z *and any of cell morphology, gene expressions, and molecular fingerprints* Y, *the encoder-based mutual information lower bound* $I_{ELB}$ *for InfoNCE can be derived by incorporating* K-1 *additional samples from the modality of* Y *(denoted as* $Y_{2:K}$) *to build the Monte Carlo estimate* m(·) *of the partition function, as follows* [34, 37]:

(4) $$I_{ELB}=1+E_{p(Z,Y)p\left(Y_{2:K}\right.}\left[log\frac{e^{h(Z,Y)}}{m\left(Z;Y,Y_{2:K}\right)}\right]-E_{p(Z)p\left(Y_{2:K}\right)p(Y)}\left[\frac{e^{h(Z,Y)}}{m\left(Z;Y,Y_{2:K}\right)}\right],$$

where h(Z,Y) is the neural network parameterized critic for density approximation with the energy-based variational family. The decoder-based lower bound $I_{DLB}$ is defined in Eq. (2), then we have that $I_{DLB}$ is tighter than $I_{ELB}$, i.e., $I(Z;Y)\geq I_{DLB}(Z;Y)\geq I_{ELB}(Z;Y)$.

Proofs are in appendix A.2. The result aligns with empirical observations in previous studies such as DALL-E 2 [40], where a prior model was introduced to improve representations from CLIP [39] before decoding to another modality. In this work, we learn decodable latent representations from molecules to align with different biological features.

## 5 Experiments

### 5.1 Implementation of Context Graph and Pretraining Setting

#### Data Source of Context Graph

We create the context graph based on (1) two Cell Painting datasets [4, 6], containing around 140K molecule perturbations (molecule and cell morphology pairs) and 15K genetic perturbations (gene and cell morphology pairs) across 1.6 billion human cells; (2) Hetionet [16], which captures gene-gene and gene-molecule relationships from millions of biomedical studies; and (3) a dataset reporting differential gene expression values for 978 landmark genes [54] for chemical perturbations (molecule and gene expression pairs) [49].

#### Node Features

Different profiling methods provide node features in different ways. Morgan fingerprints [42] are feature vectors extracted from each molecule’s structure, CellProfiler features [5] are computed from the image of each cell and represent cell morphology, and L1000 profiles [49] capture gene expression values on 978 landmark genes from cells treated with a chemical perturbation. Here are two practical considerations for the context graphs: (1) Chandrasekaran et al. [6] provided one dataset that measured the cell morphology impacts of perturbing individual genes. The 15K genetic perturbations [6] provide gene-cell morphology pairs but lack corresponding gene expression profiles. Still, we keep the gene nodes from this dataset to account for potential gene-gene interactions and incorporate cell morphology features into them. (2) All 978 landmark genes have expression values linked to the molecules used in [54]. We update new gene expression nodes with 978-dimensional feature vectors. These vectors summarize all molecule-gene expression connections for a small molecule perturbation. This approach efficiently reduces dense connections between landmark genes and molecules. We select the top 1% of gene-molecule expression values as new edges to enrich the context graph’s connectivity. We scale cell morphology and gene expression features to a range of 0 to 1 using the Min-Max scaler along each dimension.

#### Edge Weights

We assign a weight of 1 to edges defined by chemical perturbations from [4, 6]. For edges defined by computational criteria, we compute cosine similarity for nodes if they are in the same feature space (such as two cell morphology/gene expression profiles, or Morgan fingerprints). To avoid noisy edges from computations, we (1) apply a 0.8 threshold for cosine similarity, and additionally (2) explicitly enforce 99.5% sparsity by selecting top similar edges.

All together, this results in a context graph of 276,855 nodes (129,592 molecules, 4533 genes + 13,795 gene expressions, and 128,935 cell morphology) and 366,384 edges.

#### Encoder and Decoder

We use the Graph Isomorphism Model (GIN) [56] as the molecular structure encoder. All molecules on the context graph are used to pretrain the molecular representation encoder. Because we extract feature vectors as the decoding targets in different modalities, we could efficiently use Multi-Layer Perception (MLP) as modality decoders. In each training batch, random walks start from the molecule node to extract the walk path. Then, decoders are pretrained to reconstruct corresponding node features from nodes over the path. More details are in appendix B.

Next, we focus on three research questions (RQs) regarding InfoAlign’s representation for molecular property prediction, molecule-morphology matching, and hyperparameter analysis.

### 5.2 RQ1: Molecular Property Prediction

#### 5.2.1 Experimental Setting

We fine-tune MLPs on various representation learning approaches for predicting molecular properties.

##### Dataset and Evaluation.

We select datasets for important tasks in drug discovery, including activity classification for various assays in ChEMBL2K [13] and Broad6K [32], drug toxicity classification using ToxCast [41], and absorption, distribution, metabolism, and excretion (ADME) regression using Biogen3K [11]. The dataset statistics are in Table 1. We apply scaffold-splitting for all datasets. We follow [18] for the ToxCast dataset, and a 0.6:0.15:0.25 ratio for training, validation, and test sets for other datasets. We use the Area under the curve (AUC) for classification and mean absolute error (MAE) for regression. Mean and standard deviations are reported from ten runs.

##### Baseline.

We consider baselines from three representation sources: molecular structures, cell morphology, and gene expression. These expand into six categories: (1) Molecular descriptors/fingerprints (FP) of chemical structure [42]; (2) pretrained GNN representations of chemical structure from {AttrMask, ContextPred, EdgePred} [17]; (3) pretrained chemical language model (ChemLM) representations of chemical structure from {Roberta, GPT2, MolT5, ChemGPT} [12]; (4,5) cell morphology and differential gene expression values from cells treated with each molecule [54, 6]; (6) CLOOME [45] and InfoCORE [53] for multi-modal alignment, using all three data types (structure, morphology, and gene expression). We use MLPs, Random Forests (RF), and Gaussian Processes (GP) for representations for (1,4,5). We implemented up to 19 baselines, presenting the best-performing result in each of categories (1)-(5). Setting details and all results are in appendices C.1 and C.3.

#### 5.2.2 Results and Analysis

**Figure 3:**
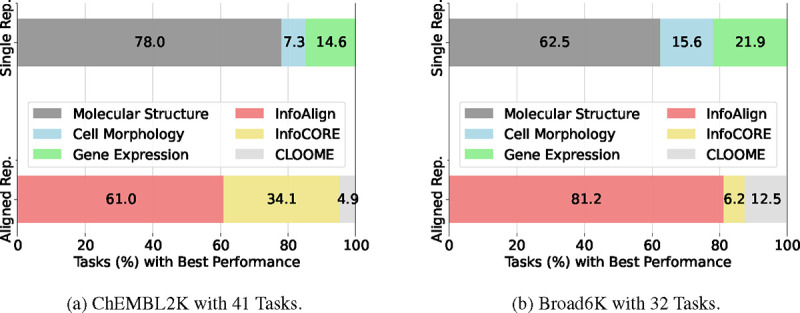
Percentage of Tasks Where Representations Excel: We compare the relative performance of three single representation (Single Rep.) approaches (molecular structure, cell morphology, and gene expression) and three aligned representations (Aligned Rep.): InfoAlign, CLOOME, InfoCORE.

Results for molecular property prediction across various assays are presented in Tables 2 and 3 and Figure 3. Results for all 19 baselines are in appendix C.3. Key observations include:

1. **Molecular structures are superior compared to cell morphology and gene expression features for predicting various molecular assays.** This is likely because the datasets and tasks we selected fundamentally involve predicting the binding affinity of a molecule to a protein [13]; furthermore, in these datasets, molecules with activity in a given assay tend to have highly related structures, rather than representing two or more structurally distinct classes of molecules with activity; together this implies that molecular structure alone will tend to yield strong results. When comparing the three popular structure-based representation approaches, fingerprints outperform the others on Broad6K and Biogen3K. Pretrained GNN and ChemLM representations excel on ChEMBL2K and ToxCast, respectively. These findings suggest that continued efforts in better structure-based representation learning are still necessary.
2. **Cell morphology and gene expression features may complement molecular structures, yielding more generalizable representations.** As shown in Figure 3, cell morphology and gene expression outperform molecular structure in approximately 20% and 36% of tasks on the ChEMBL2K and Broad6K datasets, respectively. This suggests that incorporating cell context into representation learning would be beneficial. That said, existing multi-modal baselines (InfoCORE, CLOOME) only outperform molecular structure-based approaches on ChEMBL2K and ToxCast, as they do not construct molecular representations holistically by using all cell-related modalities.
3. **InfoAlign achieves the best performance by capturing bottleneck representation across molecules, cell morphology, and gene expression.** InfoAlign improves classification and regression performance by +10.58% and +6.33% compared to the best baselines on the Broad6K and Biogen3K datasets, respectively. This improvement is even more dramatic when using the 80% AUC threshold on classification datasets. While InfoCORE (GE) is the best among baselines for the ChEMBL2K and ToxCast datasets, it cannot align the molecular representation with more than two modalities and sometimes leads to negative transfer as observed in Broad6K and Biogen3K.

### 5.3 RQ2: Molecule-Morphology Cross-Modal Matching

#### 5.3.1 Experimental Setting

We evaluated zero-shot matching performance of various methods for predicting cell morphology from query molecules, including baselines CLOOME and InfoCORE (CP) with pretrained encoders. For retrieval, we calculate the cosine similarity between the molecular representation and all cell morphology candidates, rank these candidates, and compute Normalized Discounted Cumulative Gain (NDCG) and HIT scores for the top-1 and top-10 candidates as metrics. To ensure a fair evaluation of zero-shot matching, we exclude the cell morphology data for molecules that were used to train the baseline encoders. Consequently, we have 80 molecule-cell morphology pairs from ChEMBL2K and 196 pairs from Broad6K. All the morphology data are used as candidates for matching.

For InfoAlign, we use the pretrained decoder from Section 5.1 to extract the morphology features of the encoded molecule and then calculate the likelihood of these decoded features against the candidate morphology data. We then rank the candidates in the decoding space based on their likelihood scores.

#### 5.3.2 Results and Analysis

Cross-modal matching results are in Table 4. InfoAlign outperforms InfoCORE on ChEMBL2K and is comparable to InfoCORE on Broad6K, with both surpassing CLOOME. Additionally, we visualized the distribution of ranking positions for correct matching pairs to compare overall retrieval performance. The results show that InfoAlign and InfoCORE perform similarly, while CLOOME consistently ranks correct pairs lower.

### 5.4 RQ3: Hyperparameter Analysis

**Figure 4:**
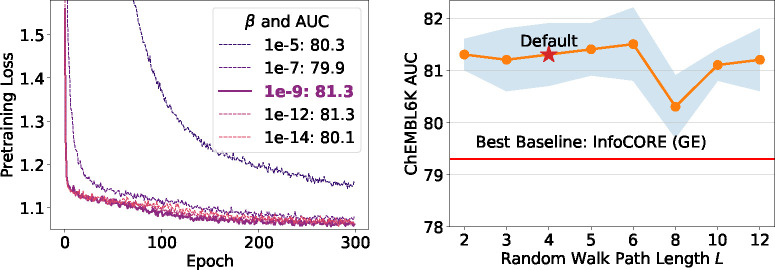
Analysis on the hyperparameters: strength of prior β and random walk length L. AUC is computed on the test set of ChEMBL2K.

Lastly, we perform analysis for the hyperparameters: the strength of the regularization to the prior Gaussian distribution β and the length of the random walk paths L. Results are presented in Figure 4. We observe a trade-off between the principles of minimality and sufficiency in Figure 4a: a too-high β value (minimal information) makes it challenging for the representation to be sufficiently expressive for molecular, gene expression, and cell morphology features, potentially degrading downstream performance. Conversely, a too-low β value weakens minimality and may impair generalization. The convergence of the pretraining loss could serve as a good indicator to balance these aspects. For the hyperparameter L, we observe in Figure 4b that downstream performance on ChEMBL2K is relatively robust across a wide range of walk lengths.

## 6 Conclusion

In this work, we proposed learning molecular representations in a cell context with three modalities: molecular structure, gene expression, and cell morphology. We introduced the information bottleneck approach, InfoAlign, using a molecular graph encoder and multiple MLP decoders. InfoAlign learned minimal sufficient molecular representations extracted by reconstructing features in the random walk path on a cellular context graph. This context graph incorporated molecules, cell morphology, and gene expression information defined in scalar or vector spaces to construct nodes, and used various chemical, biological, and computational criteria to define their weighted edges. We demonstrated the theoretical and empirical advantages of the proposed method. InfoAlign outperformed other representation learning methods in various molecular property prediction and zero-shot molecule-morphology matching tasks.

## Figures and Tables

**Table 1:** Datasets and task information. Classf. denotes classification and Regr. denotes regression.

Dataset	Type	# Task	# Molecules	# Atoms Avg./Max	# Edges Avg./Max	# Available Cell Morphology	# Available Gene Expressions

ChEMBL2K	Classf.	41	2355	23.7/61	25.6/68	2353	631
Broad6K	Classf.	32	6567	34.1/74	36.8/82	2673	1138
ToxCast	Classf.	617	8576	18.8/124	19.3/134	N.A.	N.A.
Biogen3K	Regr.	6	3521	23.2/78	25.3/84	N.A.	N.A.

**Table 2:** Results on ChEMBL2K and Broad6K. We report average AUC (Avg.), as well as the percentage of tasks achieving AUC above 80%, 85%, and 90%. **Best** and second best mean are **bold** and underline, respectively.

(# Molecule / # Task) Method	ChEMBL2K (AUC ↑) (2355 /41)				Broad6K (AUC ↑) (6567 / 32)			

	Avg.	> 80 %	> 85 %	> 90 %	Avg.	> 80 %	> 85 %	> 90 %

Morgan FP	76.8±2.2	48.8±3.9	34.6±6.3	21.9±5.7	63.3±0.3	6.3±0.0	**4.4**±1.7	**3.1**±0.0
Pretrained GNN	77.0±0.5	55.1±1.3	34.2±4.6	14.6±1.7	59.9±0.2	7.5±1.7	3.1±0.0	**3.1**±0.0
Pretrained ChemLM	74.7±1.9	46.3±3.4	35.1±4.4	22.9±1.3	60.6±0.3	7.5±1.7	3.1±0.0	1.9±1.7
Cell Morphology	64.3±2.4	15.6±6.6	8.3±3.7	4.9±3.9	55.3±0.1	0.0±0.0	0.0±0.0	0.0±0.0
Gene Expression	56.1±1.1	5.1±1.4	3.4±1.3	3.4±1.3	56.9±1.4	1.9±1.7	1.9±1.7	1.9±1.7

CLOOME	66.7±1.8	26.8±4.6	16.1±3.7	10.7±5.1	61.7±0.4	3.1±0.0	3.1±0.0	0.0±0.0
InfoCORE (GE)	79.3±0.9	62.4±2.8	46.3±3.0	30.3±2.2	60.2±0.2	3.1±0.0	0.0±0.0	0.0±0.0
InfoCORE (CP)	73.8±2.0	37.6±9.2	26.3±4.7	10.7±4.1	61.1±0.2	6.3±0.0	3.1±0.0	0.0±0.0
InfoAlign (Ours)	**81.3**±0.6	**66.3**±2.7	**49.3**±2.7	**35.1**±3.7	**70.0**±0.1	**18.8**±2.2	3.1±0.0	**3.1**±0.0

**Table 3:** Results on ToxCast and Biogen3K. We report the average AUC and the percentage of AUC above 80% on ToxCast, and regression MAE (scaled by × 100) for Biogen3K. **Best** and second best mean are **bold** and underline, respectively.

(# Molecule / # Task) Method	ToxCast (AUC ↑) (8576 / 617)		Biogen3K (MAE ×100 ↓) (3521 /6)						

	Avg.	>80 %	Avg.	hPPB	rPPB	RLM	HLM	ER	Solubility

Morgan FP	57.6±1.0	1.6±0.3	52.8±0.2	44.2±0.1	44.2±0.1	42.0±0.2	67.7±0.7	66.9±0.9	51.6±0.1
Pretrained GNN	63.5±1.1	4.8±3.0	67.3±0.3	82.4±1.1	49.8±0.7	51.7±1.0	**57.9**±0.6	62.6±0.5	99.1±1.2
Pretrained ChemLM	64.7±0.9	3.6±1.1	65.1±0.5	76.7±2.1	55.9±1.1	49.2±1.0	70.3±0.8	73.1±1.0	65.3±1.7

CLOOME	54.2±0.9	0.9±0.2	64.3±0.4	65.2±1.5	56.9±0.8	44.2±0.8	70.7±0.4	73.6±0.8	75.0±2.1
InfoCORE (GE)	65.3±0.2	5.4±1.7	69.9±1.2	79.9±3.6	51.6±1.8	51.3±2.1	78.6±0.3	77.8±1.9	80.3±0.9
InfoCORE (CP)	62.4±0.4	1.3±0.5	71.0±0.6	74.5±4.9	53.5±0.7	53.6±2.1	80.8±1.5	79.4±3.4	84.4±1.0
InfoAlign (Ours)	**66.4**±1.1	**6.6**±1.6	**49.4**±0.2	**39.7**±0.4	**39.2**±0.3	**40.5**±0.6	66.7±1.7	**62.0**±1.5	**48.4**±0.6

**Table 4:** Retrieval results on ChEMBL2K (top) and Broad6K (bottom): Left tables display ranking metrics for top candidates. Right figures visualize the distribution of rankings for the correct matching.

ChEMBL2K	NDCG % (↑)		HIT % (↑)		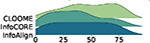

	top-1	top-10	top-1	top-10	
	
CLOOME	0	2.0	0	6.3	
InfoCORE	0	4.5	0	11.3	
InfoAlign	**1.3**	**5.7**	**1.3**	**12.5**	
	
Broad6K	NDCG % (↑)		HIT % (↑)		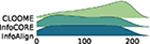

	top-1	top-10	top-1	top-10	
	
CLOOME	0.5	0.9	0.5	1.5	
InfoCORE	**1.0**	**2.5**	**1.0**	**4.6**	
InfoAlign	0.5	2.3	0.5	5.1	

**Figure 5:**
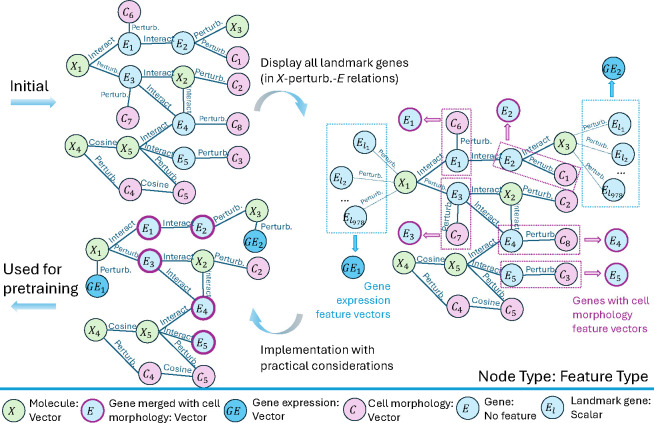
From the initial idea in Section 4 to the practical implementation of the context graph, we first display relations between molecules and all the landmark genes from [54] for the $X_{1}-E_{3}$ and $X_{3}-E_{2}$ relationships. $E_{3}$ and $E_{2}$ are landmark genes involved in small molecule perturbations and cell morphology perturbation; we display them separately for clarity. Next, we merge all landmark genes into new gene expression nodes and integrate genes from genetic perturbations in the JUMP dataset [6] with cell morphology features. Practical considerations are detailed in Section 5.1 and appendix B.

**Figure 6:**
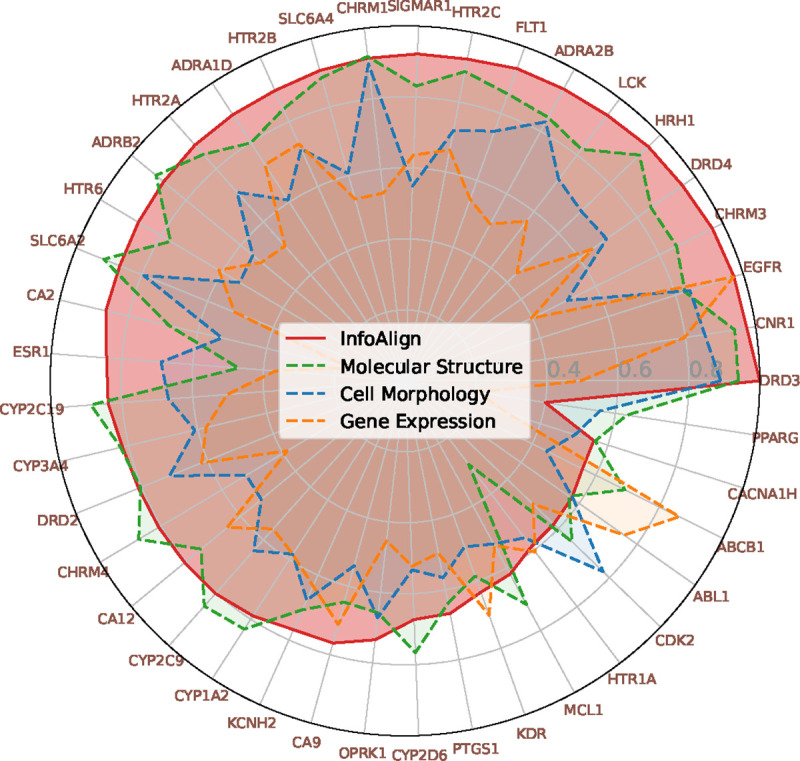
An overview of representation’s predictive performance on all 41 bioactivity prediction tasks on ChEMBL2K.

**Figure 7:**
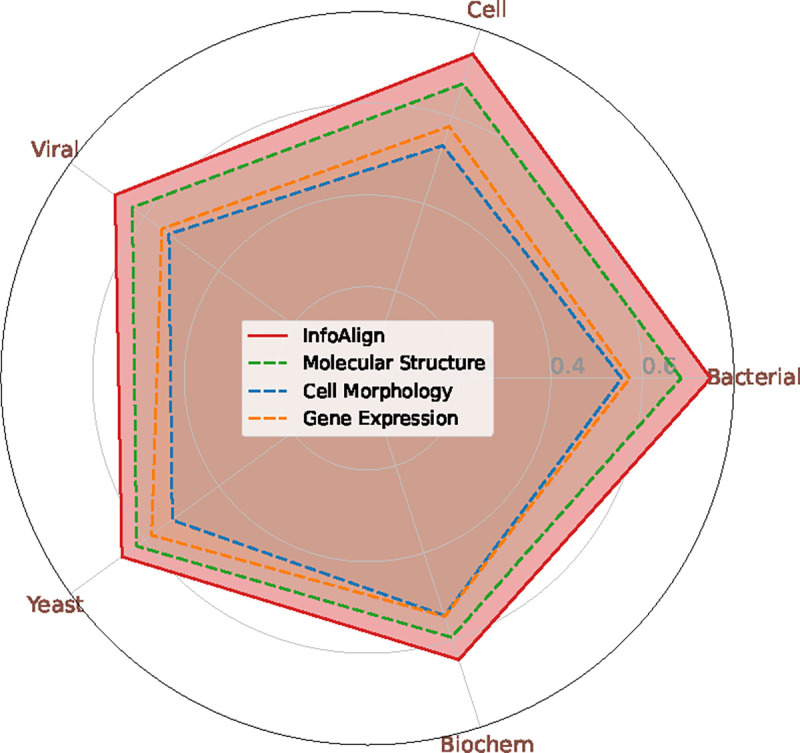
An overview of representation’s predictive performance on five major task categories on Broad6K.

## A Proof Details

### A.1 Proof of Eq. (2)

For the molecule X, its latent representation Z, and any biological modality variable Y, the exact computation of I(Z;Y) and I(X;Z) is intractable due to the unknown conditional distribution p(Y∣Z) and the marginal p(Z). We introduce the variational approximations q(Y∣Z) for a lower bound on I(Z;Y):

(5)
$$I(Z;\;Y)\;=E_{p(Z,Y)}\left[log\frac{p(Z,\;Y)q(Y|Z)}{p(Y)p(Z)q(Y|Z)}\right],\;\;=E_{p(Z,Y)}\left[log\frac{q(Y|Z)}{p(Y)}\right]+E_{p(Z)}\left[\mathrm{KL}\left(p\left(Y|Z\right)∥q\left(Y|Z\right)\right)\right],\;\;\geq E_{p(Z,Y)}[log\;q(Y|Z)]+H(Y)≜I_{DLB}$$

This is because that KL(p(Y∣Z)∥q(Y∣Z))≥0. We introduce the variational approximations q(Z) for a upper bound on I(X;Z):

(6)
$$I(X;\;Z)\;=E_{p(X,Z)}\left[log\frac{p(X,\;Z)q(Z)}{p(X)p(Z)q(Z)}\right],\;\;=E_{p(X,\;Z)}\left[log\frac{p(Z|X)}{q(Z)}\right]-E_{p\left(Z\right)}[\left(p\left(Z\right)∥q\left(Z\right)\right],\;\;\leq E_{p(X)}[KL(p(Z|X)∥q(Z))]≜I_{EUB}$$

### A.2 Proof of proposition 4.2

For the molecular representation Z, and any biological modality variable Y, we use the neural network parameterized critic h(Z,Y) with the energy-based variational family for density approximation [37]:

$$q\left(Y|Z\right)=\frac{p\left(Y\right)}{E_{p\left(Y\right)}\left[e^{h\left(Z,Y\right)}\right]}e^{h\left(Z,Y\right)}.$$

Thus, we can rewrite $I_{DLB}$ based on the unnormalized distribution of q(Y∣Z):

(7)
$$I_{DLB}\;=E_{p(Z,Y)}[log\;q(Y|Z)]+H(Y)\;\;=E_{p(Z,Y)}\left[log\left(\frac{p(Y)}{E_{p(Y)}\left[e^{h(Z,Y)}\right]}e^{h(Z,Y)}\right)\right]-E_{p\left(Y\right)}\left[\log p\left(Y\right)\right],\;\;=E_{p(Z,Y)}\left[h\left(Z,Y\right)\right]-E_{p\left(Z,Y\right)}\left[E_{p\left(Y\right)}\left[e^{h\left(Z,Y\right)}\right]\right],\;\;=E_{p(Z,Y)}\left[h\left(Z,Y\right)\right]-E_{p\left(Z\right)}\left(\log\tilde{Z}\left(Z\right)\right),$$

where $\tilde{Z}(Z)=E_{p(Y)}\left[e^{h(Z,Y)}\right]$ is the partition function.

Note that the log partition function is intractable. Poole et al. [37] introduced a new variational parameter a(·) to upper bound $\tilde{Z}(Z)$, deriving a tractable lower bound for $I_{DLB}$:

(8)
$$I_{DLB}\geq E_{p\left(Z,Y\right)}\left[h\left(Z,Y\right)\right]-E_{p\left(Z\right)}\left[\frac{E_{p\left(Y\right)}[e^{h\left(Z,Y\right)}]}{a\left(Z\right)}+\log\left(a\left(Z\right)\right)-1\right].$$

This is because ∀x,a>0, the inequality $log(x)\leq \frac{x}{a}+log(a)-1$ holds, which can be applied to the second term of Eq. (7). The $I_{NWJ}$ bound [34] is a special case where a(Z)=e.

(9)
$$I_{NWJ}\;≜E_{p(Z,Y)}[h(Z,Y)]-E_{p(Z)}\left[\frac{E_{p(Y)}\left[e^{h(Z,Y)}\right]}{e}+log(e)-1\right]\;\;=E_{p(Z,Y)}\left[h\left(Z,Y\right)\right]-e^{-1}E_{p\left(Z\right)}\left[\tilde{Z}\left(Z\right)\right].$$

$I_{NWJ}$ has high variance due to the estimation of the upper bound on the log partition function. Based on $I_{NWJ}$ and multiple examples, one can derive the encoder-based lower bound $I_{ELB}$ for InfoNCE.

Suppose there are K-1 additional examples independently and identically sampled from the modality of X, denoted as $Y_{2:K}$, and the critic is configured with parameters a(⋅) as $1+log\frac{e^{h(Z,Y)}}{a\left(Z;Y,Y_{2:K}\right)}$. Then, we can rewrite $I_{NWJ}$ for its multi-sample version:

(10)
$$I_{NWJ}\;=E_{p(Z,Y)p\left(Y_{2:K}\right)}\left[1+log\frac{e^{h(Z,Y)}}{a\left(Z;Y,Y_{2:K}\right)}\right]-e^{-1}E_{p\left(Y\right)p\left(Z\right)p\left(Y_{2:K}\right)}\left[e^{1+\log\frac{e^{h\left(Z,Y\right)}}{a\left(Z,Y,Y_{2:K}\right)}}\right],\;\;=1+E_{p(Z,Y)p\left(Y_{2:K}\right)}\left[log\frac{e^{h(Z,Y)}}{a\left(Z;Y,Y_{2:K}\right)}\right]-E_{p\left(Y\right)p\left(Y_{2:K}\right)p\left(Z\right)}\left[\frac{e^{h\left(Z,Y\right)}}{a\left(Z;Y,Y_{2:K}\right)}\right].$$

Multiple samples can be utilized for the Monte Carlo method $m\left(Z;Y,Y_{2:K}\right)$ to estimate the upper bound on the partition function $a\left(Z;Y,Y_{2:K}\right)$:

$$a\left(Z;Y,Y_{2:K}\right)=m\left(Z;Y,Y_{2:K}\right)=\frac{1}{K}\left(e^{h(Z,Y)}+\sum_{i=2}^{K}e^{h\left(Z,Y_{i}\right)}\right),$$

where K-1 independent samples are drawn from $\prod_{i}p\left(Y_{i}\right)$ and one sample from p(Z,Y) for the term $E_{p(Z,Y)p\left(Y_{2:K}\right)}[\cdot ]$ or K samples from $\prod_{i=1}^{K}p\left(Y_{i}\right)$ (we set $Y_{1}=Y$) for a p(Z) sample in the $E_{p(Y)p\left(Y_{2:K}\right)}[\cdot ]$ term. Therefore, we can derive $I_{ELB}≜I_{NCE}$:

(11)
$$I_{ELB}≜I_{NCE}=1+E_{p(Z,Y)p\left(Y_{2:K}\right)}\left[\log\frac{e^{h(Z,Y)}}{m\left(Z;Y,Y_{2:K}\right)}\right]-E_{p(Y)p\left(Y_{2:K}\right)p(Z)}\left[\frac{e^{h(Z,Y)}}{m\left(Z;Y,Y_{2:K}\right)}\right],\;\;\;\;\;\;\;\;\;\;\;=1+E_{p\left(Y|Z\right)p\left(Z\right)p\left(Y_{2:K}\right)}\left[\text{log}\frac{e^{h\left(Z,Y\right)}}{\frac{1}{K}\sum_{i=1}^{K}e^{h\left(Z,Y_{i}\right)}}\right]\;\;\;\;\;\;\;\;\;\;\;\;\;\;\;-E_{p\left(Y\right)p\left(Y_{2:K}\right)p\left(Z\right)}\left[\frac{e^{h\left(Z,Y\right)}}{\frac{1}{K}\sum_{i=1}^{K}e^{h\left(Z,Y_{i}\right)}}\right],\;\;\;\;\;\;\;\;\;\;\;=E_{p(Z,Y)}[h(Z,Y)]-E_{p(Z)}\left[\log\frac{1}{K}\sum_{i=1}^{K}e^{h\left(Z,Y_{i}\right)}\right].$$

Note that for $E_{p(Y)p\left(Y_{2:K}\right)p(Z)}[\cdot ]$, we average the bound over K replicates as well to ensure that the last term in Eq. 10 is the constant 1. Now, $I_{ELB}$ or $I_{NCE}$ is upper bounded by logK, rather than a(⋅). Hence, the difference between $I_{DLB}$ and $I_{ELB}$ is

(12)
$$I_{DLB}-I_{ELB}=E_{p\left(Z\right)}\left[\text{log}\frac{1}{K}\sum_{i=1}^{K}e^{h\left(Z,Y_{i}\right)}\right]-E_{p\left(Z\right)}\left(\text{log}\;\tilde{Z}\left(Z\right)\right)\geq 0.$$

When K is sufficiently large to estimate the partition function, we have $E_{p(Z)\;}\left[log\left(E_{p(Y)}\left[e^{h(Z,Y)}\right]\right)\right]$ for the left term, indicating that $I_{DLB}-I_{ELB}=0$. Since $I_{NCE}$ is upper bounded by logK [35], smaller values of K may result in a less tight $I_{ELB}$, causing $I_{DLB}-I_{ELB}\geq 0$ to always hold. In particular, I(Z;Y)>logK implies that the bound $I_{ELB}$ will be loose.

## B Context Graph Details

### B.1 Edge Construction

Edges represent similarity relationships between molecules, genes and cells. According to the chemical or biological criteria, we have following types of edges:

1. $e\in {𝒴}^{\left[X\right]}\times {𝒴}^{\left[C\right]}$: These edges are introduced through molecule perturbation experiments from cell painting datasets created by [3] and the JUMP dataset [6]. It links molecule nodes with cell morphology nodes. We use the edge weight 1 for all these edges.
2. $e\in {𝒴}^{\left[E\right]}\times {𝒴}^{\left[C\right]}$: These edges should be introduced by genetic perturbation from the JUMP dataset [6]. The perturbations are either based on gene overexpression (ORF) or gene knockout techniques (CRISPR). They link the gene nodes and the cell morphology nodes. However, the genes introduced by the genetic perturbations lack gene expression profiling from [49] as node features. We did not implement gene-cell morphology edges from [6] due to the absence of differential gene expression profiling values [49]. Instead, we merged the gene nodes from [6] with their linked cell morphology nodes, creating single nodes. This approach enables a more efficient context graph, incorporating some gene nodes with cell morphology features.
3. $e\in {𝒴}^{\left[X\right]}\times {𝒴}^{\left[E\right]}$: These edges could represent molecule-gene binding and regulation relationships, linking molecules to genes [16]. Some links can be sourced from [16], and we also retrieve gene-molecule links from [54] by selecting the top 5% absolute differential expression values.
4. $e\in {𝒴}^{\left[E\right]}\times {𝒴}^{\left[E\right]}$: These edges denote the relationships of gene-gene covariance and interaction and we use the links from [16].

We enrich the edges in the context graph by incorporating computational similarity edges, where cosine similarity is computed among within nodes having the same type and feature vectors. We note that the cell morphology features from [3] and [6] have different dimensions since the latter has applied batch correction techniques [2] on the CellProfiler features [5]. Thus, we cannot compute the similarity between these two subsets of cell morphology nodes. We use (1) a 0.8 similarity threshold and (2) a minimal sparsity of 99.5% by selecting top 0.5% similar edges to avoid excessive noise in computational similar edges.

### B.2 Dataset Sources of Nodes

Here are the datasets we used to create different types of nodes on the context graph:

**Table 5:** Complete results on ChEMBL2K. We report average AUC (Avg.), as well as the percentage of tasks achieving AUC above 80%, 85%, 90% and 95%. The best results in each category are  and reported in Table 2.

Repr.	Model	Avg.	> 80 %	> 85 %	> 90 %	> 95%

	MLP	76.8±2.2	48.8±3.9	34.6±6.3	21.9±5.7	12.2±2.4
Morgan FP	RF	54.7±0.7	0.0±0.0	0.0±0.0	0.0±0.0	0.0±0.0
	GP	51.0±0.0	0.0±0.0	0.0±0.0	0.0±0.0	0.0±0.0

	AttrMask	74.0±0.5	46.8±2.7	31.2±4.4	14.6±1.7	2.4±0.0
Pretrained GNN	ContextPred	77.0±0.5	55.1±1.3	34.2±4.6	14.6±1.7	1.5±1.3
	EdgePred	75.6±0.5	54.1±4.0	34.6±7.2	12.2±2.4	6.3±1.3

	Roberta-102M	74.7±1.9	46.3±3.5	35.1±4.4	22.9±1.3	7.8±3.2
Pretrained ChemLM	GPT2–87M	71.0±3.4	31.2±11.2	20.0±9.4	7.3±6.9	2.9±3.2
	MolT5	70.0±0.8	32.2±2.0	21.0±4.1	8.8±1.3	3.4±1.3
	ChemGPT-19M	65.0±1.1	16.1±2.8	11.2±3.3	5.4±1.1	2.9±1.1

	MLP	64.3±2.4	15.6±6.6	8.3±3.7	4.9±3.9	2.9±2.7
Cell Morphology	RF	55.9±0.7	3.9±1.3	3.9±1.3	2.4±0.0	2.4±0.0
	GP	51.5±0.0	0.0±0.0	0.0±0.0	0.0±0.0	0.0±0.0

	MLP	56.1±1.1	5.1±1.4	3.4±1.3	3.4±1.3	2.4±0.0
Gene Expression	RF	52.8±0.3	0.0±0.0	0.0±0.0	0.0±0.0	0.0±0.0
	GP	Run out of time				

Aligned	CLOOME	66.7±1.8	26.8±4.6	16.1±3.7	10.7±5.1	3.4±2.8
	InfoCORE (GE)	79.3±0.9	62.4±2.8	46.3±3.0	30.3±2.2	18.1±1.3
	InfoCORE (CP)	73.8±2.0	37.6±9.2	26.3±4.7	10.7±4.1	5.9±2.2
	InfoAlign	81.3±0.6	66.3±2.7	49.3±2.7	35.1±3.7	18.5±2.8

- Molecule nodes: Molecular nodes are sourced from two cell painting datasets: one by Bray et al. [4] and the other from the recently released JUMP dataset [6], and the third source from Wang et al. [54], which are used to study adverse drug reactions.
- Gene nodes: Gene nodes are from the landmark genes used by Wang et al. [54] in creating the LINCS L1000 profiling of drugs. Other gene nodes come from genetic perturbations in the JUMP dataset [6]. The gene nodes from [6] have cell morphology features as described in appendix B.1. The landmark gene nodes from [54] have scalar gene expression profiles, but these values are updated in the new gene expression nodes.
- Cell morphology nodes: Cell nodes are sourced from the two cell painting datasets [3, 6].
- Gene expression nodes: Based on landmark genes from [54], each gene expression node summarizes all gene expression profiles into vectors from a small molecule perturbation. Since Wang et al. [54] measured the same landmark genes for a set of molecules, we update new gene expression nodes with feature vectors for all these landmark genes. This approach efficiently constructs decoding targets from molecules to gene expression profiles and prevents redundant gene-molecule connections.

We present an example of the cellular context graph in Figure 5.

## C Experiment Details

### C.1 Prediction Datasets

All experiments were run on a single 32G V100. Prediction dataset statistics are in Table 1:

- ChEMBL2K [13]: The dataset is a subset of the ChEMBL dataset [13], overlapping with the JUMP CP [6] datasets. We determined activity using the “activity_comment” provided by ChEMBL. If not, we applied a threshold of 6.5, labeling compounds with pChEMBL > 6.5 as active. We exclude all molecules in the dataset from the pretraining set to avoid data leakage. There are a total of 41 tasks related to protein binding affinity, which are converted to binary activity values. We filter the dataset to ensure that each task has at least one positive and five negative examples.

- Broad6K [32]: The original version provided by Moshkov et al. [32] is a collection of 16,170 molecules tested in 270 assays, resulting in a total of 585,439 readouts. However, there are a large number of missing values, with 153 assays having a missing value percentage above 99%. To mitigate bias in the conclusions, we extract subsets where the percentage is less than 50%.
- ToxCast [41]: The toxicology data is collected from the “Toxicology in the 21st Century” initiative, widely utilized in many graph machine learning models [18]. The dataset comprises 8,576 molecules and 617 binary classification tasks.
- Biogen3K [11]: The dataset includes properties that describe the disposition of a drug in the body, including absorption, distribution, metabolism, and excretion (ADME). It is collected from 120 Biogen datasets across six ADME in vitro endpoints over 20 time points spanning about 2 years. The endpoints include human liver microsomal (HLM) stability reported as intrinsic clearance (Clint, mL/min/kg), MDR1-MDCK efflux ratio (ER), Solubility at pH 6.8 (μg/mL), rat liver microsomal (RLM) stability reported as intrinsic clearance (Clint, mL/min/kg), human plasma protein binding (hPPB) percent unbound, and rat plasma protein binding (rPPB) percent unbound.

We utilize scaffold-splitting with a ratio of 0.6:0.15:0.25 and follow [18] for the ToxCast dataset. We use the Area under the curve (AUC) score for classification and mean absolute error (MAE) for regression. We report the mean and standard deviations from ten runs.

### C.2 Implementation and Baseline

We consider baselines from three representation sources: molecular structures, cell morphology, and gene expressions. Moreover, we have three different ways to represent molecular structures, including fingerprints based on domain knowledge, GNNs based on the graph structure of molecules, and chemical language models (ChemLM) based on SMILES-sequence structure of molecules.

**Table 6:** Complete results on Broad6K. We report average AUC (Avg.), as well as the percentage of tasks achieving AUC above 80%, 85%, 90% and 95%. The best results in each category are  and reported in Table 2.

Repr.	Model	Avg.	> 80 %	> 85 %	> 90 %	> 95%

	MLP	63.3±0.3	6.3±0.0	4.4±1.7	3.1±0.0	3.1±0.0
Morgan FP	RF	55.5±0.1	0.0±0.0	0.0±0.0	0.0±0.0	0.0±0.0
	GP	50.6±0.0	0.0±0.0	0.0±0.0	0.0±0.0	0.0±0.0

	AttrMask	59.8±0.2	3.1±0.0	3.1±0.0	3.1±0.0	0.0±0.0
Pretrained GNN	ContextPred	60.0±0.2	7.5±1.7	3.1±0.0	3.1±0.0	0.0±0.0
	EdgePred	59.9±0.2	3.1±0.0	3.1±0.0	3.1±0.0	0.0±0.0

	Roberta-102M	59.8±0.7	5.0±1.7	3.1±0.0	3.1±0.0	0.0±0.0
Pretrained ChemLM	GPT2–87M	60.6±0.3	7.5±1.7	3.1±0.0	1.9±1.7	0.0±0.0
	MolT5	56.4±0.8	3.8±1.4	2.5±1.4	2.5±1.4	0.0±0.0
	ChemGPT-19M	55.1±0.9	3.1±0.0	3.1±0.0	1.3±1.7	0.0±0.0

	MLP	51.9±1.0	0.0±0.0	0.0±0.0	0.0±0.0	0.0±0.0
Cell Morphology	RF	55.3±0.1	0.0±0.0	0.0±0.0	0.0±0.0	0.0±0.0
	GP	54.7±0.0	0.0±0.0	0.0±0.0	0.0±0.0	0.0±0.0

	MLP	56.9±1.4	1.9±1.7	1.9±1.7	1.9±1.7	0.0±0.0
Gene Expression	RF	55.2±0.2	0.0±0.0	0.0±0.0	0.0±0.0	0.0±0.0
	GP	50.1±0.0	0.0±0.0	0.0±0.0	0.0±0.0	0.0±0.0

Aligned	CLOOME	61.7±0.5	3.1±0.0	3.1±0.0	0.0±0.0	0.0±0.0
	InfoCORE (GE)	60.2±0.2	3.1±0.0	0.0±0.0	0.0±0.0	0.0±0.0
	InfoCORE (CP)	61.1±0.2	6.3±0.0	3.1±0.0	0.0±0.0	0.0±0.0
	InfoAlign	69.9±0.1	18.8±2.2	3.1±0.0	3.1±0.0	1.3±1.7

1. Molecular descriptors/fingerprints [42] (Structure only): We train MLPs, Random Forests (RF), and Gaussian Processes (GP) on these representations.
2. Pretrained GNN representations [17] (Structure only): We consider AttrMask, ContextPred, and EdgePred with supervised pretraining [17] and apply fine-tuned MLPs on top of the pretrained representations.
3. Pretrained ChemLM representations [12] (Structure only): We consider pretrained models such as 102M Roberta, 87M GPT2, MolT5, and 19M ChemGPT [12]. We apply fine-tuned MLPs on top of these pretrained representations.
  **Table 7:** Complete results on ToxCast. We report average AUC (Avg.), as well as the percentage of tasks achieving AUC above 80%, 85%, 90% and 95%. The best results in each category are  and reported in Table 3.
  Representation	Models	Avg.	>80 %	>85 %	>90 %	>95 %
  	MLP	57.6±1.0	1.6±0.3	0.6±0.4	0.3±0.3	0.0±0.0
  Morgan FP	RF	52.3±0.1	0.2±0.1	0.1±0.1	0.0±0.1	0.0±0.0
  	GP	Run out of time				
  	AttrMask	63.1±0.8	3.2±1.2	0.8±0.3	0.2±0.1	0.0±0.1
  Pretrained GNN	ContextPred	63.0±0.6	3.3±1.3	0.4±0.4	0.0±0.0	0.0±0.0
  	EdgePred	63.5±1.1	4.8±3.0	0.8±0.4	0.1±0.1	0.0±0.0
  	Roberta-102M	64.2±0.8	3.1±1.8	0.9±0.6	0.2±0.2	0.0±0.1
  Pretrained ChemLM	GPT2–87M	61.5±1.1	2.4±0.6	0.8±0.3	0.3±0.1	0.0±0.1
  	MolT5	64.7±0.9	3.6±1.1	1.1±0.3	0.4±0.2	0.0±0.1
  	ChemGPT-19M	Token Error				
  Aligned	CLOOME	54.2±1.0	0.9±0.2	0.3±0.1	0.1±0.1	0.0±0.0
  	InfoCORE (GE)	65.3±0.2	5.4±1.7	1.2±0.4	0.3±0.1	0.0±0.0
  	InfoCORE (CP)	62.4±0.4	1.3±0.5	0.3±0.3	0.0±0.0	0.0±0.0
  	InfoAlign	66.4±1.1	6.6±1.6	2.1±0.5	0.7±0.3	0.1±0.1
  **Table 8:** Complete results on Biogen3K. We report the average MAE and MAE for all tasks (scaled by × 100). The best results in each category are  and reported in Table 3.
  Representation	Models	Avg.	hPPB	rPPB	RLM	HLM	ER	Solubility
  	MLP	66.2±2.4	66.1±2.6	56.8±2.3	56.5±4.2	74.6±6.2	73.7±7.3	69.5±3.0
  Morgan FP	RF	52.8±0.2	44.2±0.1	44.2±0.1	41.9±0.2	67.7±0.7	66.9±0.9	51.6±0.1
  	GP	60.0±0.0	51.3±0.0	59.5±0.0	49.7±0.0	68.8±0.0	69.3±0.0	61.6±0.0
  	AttrMask	67.3±0.3	82.4±1.1	49.8±0.7	51.7±1.0	57.9±0.6	62.6±0.5	99.1±1.2
  Pretrained GNN	ContextPred	68.5±0.9	85.0±7.9	49.7±0.4	55.1±2.7	61.4±1.8	63.1±0.5	96.5±3.7
  	EdgePred	67.8±0.9	81.2±10.2	48.0±0.5	53.5±2.8	62.2±1.8	62.9±0.7	99.1±6.9
  	Roberta-102M	69.0±2.6	71.4±14.5	65.1±19.2	63.7±24.6	67.5±5.2	69.9±4.9	76.7±13.2
  Pretrained ChemLM	GPT2–87M	73.9±8.5	65.4±12.9	73.1±20.8	54.1±12.9	83.2±21.5	86.1±19.8	81.8±25.5
  	MolT5	65.1±0.5	76.7±2.1	55.9±1.1	49.2±1.0	70.3±0.8	73.1±1.0	65.3±1.7
  	ChemGPT-19M	75.7±8.5	59.5±7.3	88.8±32.3	76.1±11.8	84.0±20.6	77.2±8.5	68.6±7.1
  Aligned	CLOOME	64.3±0.4	65.2±1.5	56.9±0.8	44.2±0.8	70.7±0.4	73.6±0.8	75.0±2.1
  	InfoCORE (GE)	69.9±1.2	79.9±3.6	51.6±1.8	51.3±2.1	78.6±0.3	77.8±1.9	80.4±0.9
  	InfoCORE (CP)	71.0±0.6	74.5±4.9	53.5±0.7	53.6±2.1	80.8±1.5	79.4±3.4	84.4±1.0
  	InfoAlign	49.4±0.2	39.7±0.4	39.2±0.3	40.5±0.6	66.7±1.7	62.0±1.5	48.4±0.6
4. Cell Morphology [42] (Cell or Structure only): Cell morphology features are available in for part of molecules in the ChEMBL2K and Broad6K datasets. We train MLPs, RF, and GP on these representations. Note that not all molecules have corresponding cell morphology feature vectors; in such cases, we replace the predictions on the missing feature with ML predictions on the structure.
5. Gene Expression [42] (Gene or Structure only): Differential gene expression values are available for part of molecules in the ChEMBL2K and Broad6K datasets. We train MLPs, RF, and GP on these representations. Note that not all molecules have corresponding gene expression vectors over landmark genes; in such cases, we replace the predictions on the missing feature with ML predictions on the structure.
6. CLOOME [45] and InfoCORE [53] (Structure-Cell or Structure-Gene aligned): CLOOME utilizes ResNet [15] and descriptor-based MLP to align representation from cell morphology images with the molecular structure representation. We use their pretrained MLP to obtain molecular representations and fine-tune another MLP on top of these representations. InfoCORE has two versions, InfoCORE-CP and InfoCORE-GE, which align the molecular graph representation with cell morphology features or differential gene expression features, respectively. We use both versions as baselines and fine-tune another MLP on top of these representations.

### C.3 More Results for Molecular Property Prediction

We present comprehensive results for the ChEMBL2K dataset, including 19 baseline performances in Table 5 and comparisons of basic representation approaches with the InfoAlign across all task dimensions in Figure 6. Similarly, results for the Broad6K dataset, comparing InfoAlign against 19 baselines are in Table 6 and comparison with basic representations across five major task dimensions (Cell, Yeast, Viral, Biochem, and Bacterial related targets) are in Figure 7. Results for the ToxCast and Biogen3K datasets are provided in Table 7 and Table 8, respectively. From these detailed results we have more observations:

1. Different structure-based molecular representations vary in sensitivity to model architecture. Dramatic performance drops occur with Morgan FP when replacing the MLP architecture with RF or GP in the ChEMBL2K and Broad6K datasets. Conversely, in the Biogen3K dataset, RF and GP significantly outperform MLP. In contrast, pretrained GNN and ChemLM representations maintain more consistent performance across various datasets.
2. Learning universal molecular representations solely from molecular structures remains challenging, even within the representation category. For pretrained GNN representations, ContextPred outperforms others in the ChEMBL2K and Broad6K datasets but not in other datasets. For ChemLM representations, MolT5 excels over other sequential-based models in the ToxCast and Biogen3K datasets, but this is not the case with the ChEMBL2K and Broad6K datasets. Different datasets may emphasize varied aspects of bioactivity classification or regression and pose generalization challenges for molecular representation learning.
3. InfoAlign shows strong generalization for the targets of non-human cells, as shown in Figure 7. Although the context graph primarily uses data from small molecule and genetic perturbation datasets [3, 6] focused on human cell cultures, InfoAlign also exhibits robust generalization to bacterial and viral targets compared to basic representation approaches.
